# Supplementary material for: Molecular Epidemiology of Group B Streptococci in Lithuania Identifies Multi-Drug Resistant Clones and Sporadic ST1 Serotypes Ia and Ib
Source: Pathogens. 2022 Sep 17;11(9):1060. doi: 10.3390/pathogens11091060 (PMC9504518; doi:10.3390/pathogens11091060)

Supplementary Materials

Molecular epidemiology of Group B streptococci in Lithuania identifies multi-drug resistant clones and sporadic ST1 serotypes Ia and Ib

Jonah Rodgus [1,2,3,†], Ruta Prakapaite [4,5,†,‡], Panagiotis Mitsidis [1,2], Ramune Grigaleviciute [6], Rita Planciuniene [7], Povilas Kavaliauskas [4,8,9,‡] and Elita Jauneikaite [1,3,*, ‡]

(please see excel) Table S1. Summary of the genomic, demographic, and phenotypic characteristics of GBS isolates from Lithuania. This table summarises information on 42 GBS isolates from Lithuania collected between July and September 2020. Clonal Complexes (CC) were assigned using the PubMLST database. The presence of acquired antimicrobial resistance genes is indicated with the gene name. Specific amino acid changes for genes *pbp1a*, *pbp2X*, *gyrA*, and *parC* are indicated. Antimicrobial susceptibility testing results (measured mm shown in brackets) were recorded following EUCAST v.10 guidelines: S (sensitive), R (resistant). Surface genes *alpha-C*, *alp1*, *alp2/3*, *rib*, *srr1*, *srr2*, *PI-1*, *PI-1B*, *PI-2A* (all four variants), *PI-2B*, and *hvgA* were recorded as present if they had >95% sequence identity with the query gene. Hits of < 95% were also noted in the table, but not reported as positive results. Empty cells indicate that no gene or amino acid changes were present, or that no antimicrobial susceptibility test was carried out.

(please see excel) Table S2. A list of nine serotype Ia ST1 genomes downloaded from the PubMLST database for comparison analysis with ST1/CC1 genomes from this study. Metadata reported as per information available on the PubMLST database.

Figure S1. A maximum-likelihood phylogenetic tree and comparison of recombination regions of CC1 isolates. a) Tree was constructed from a recombination-free alignment of core-genome SNPs. SNPs were mapped and called using reference sequence SS1 (NCBI accession number: CP010867), representing serotype V ST1. Branch symbol colour shows the serotype of each isolate: Ia (blue), Ib (purple), and V (yellow). Tree scale indicates nucleotide substitution rate per site. **b)** BRIG was used to show predicted recombination regions among ST1 isolates of serotype Ib (LTS066), Ia (LTS045 and LTS018), and the serotype V reference SS1 (CP010867.1). From inner to outermost circle: inner circle (black line) is reference SS1, then polymorphisms identified in each of the GBS isolates: serotype Ib ST1 isolate LTS066 (red), serotype Ia ST1387 (CC1) isolate LTS045 (blue), and serotype Ia ST1 isolate LTS018 (black). Outermost circle indicates genome landmarks in reference genome SS1 as follows: cps locus - capsular polysaccharide locus, *alp*3 - alpha like surface protein encoding gene, seven multi-locus housekeeping genes (*adhP*, *atr*, *tkt*, *glcK*, *sdhA*, *glnA*, and *pheS*), MGE - mobile genetic element and pilus island genes *PI-1* and *PI-2a* (in black).

a)

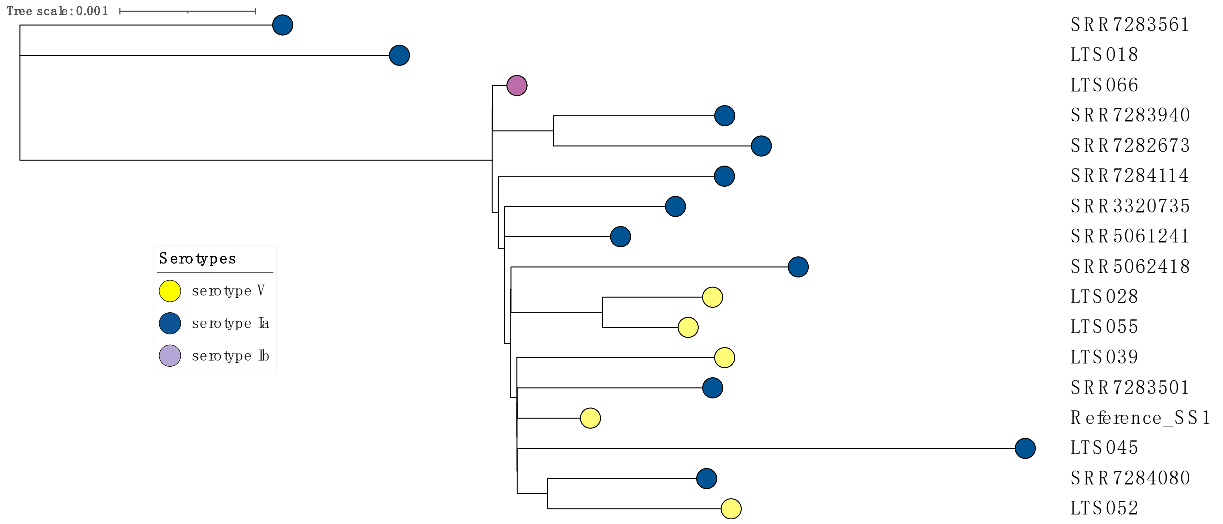
Tree scale: 0.001
Serotypes
serotype V
serotype Ia
serotype Ib
SRR7283561
LTS018
LTS066
SRR7283940
SRR7282673
SRR7284114
SRR3320735
SRR5061241
SRR5062418
LTS028
LTS055
LTS039
SRR7283501
Reference_SS1
LTS045
SRR7284080
LTS052

b)

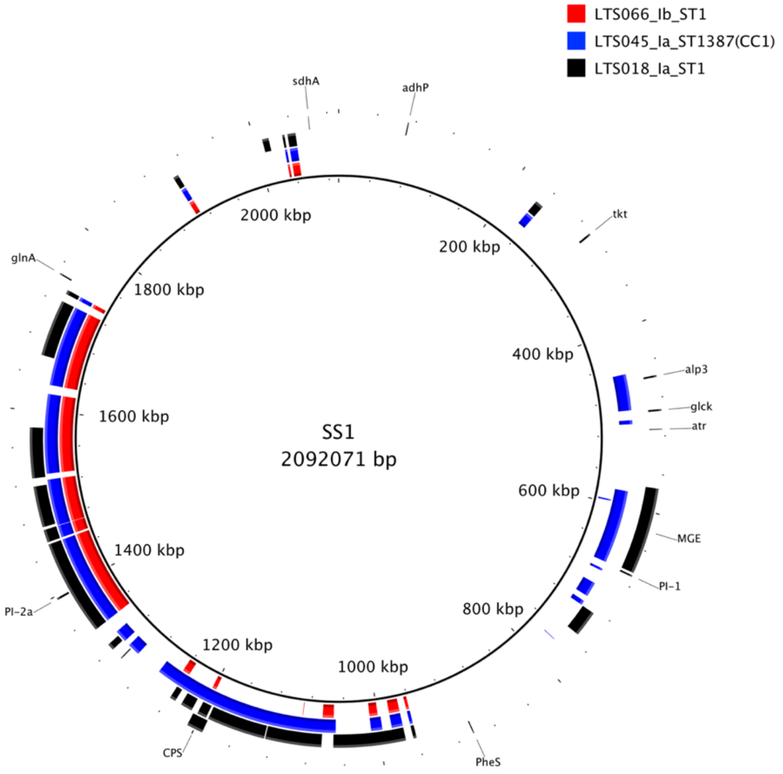
LTS066_Ib_ST1
LTS045_Ia_ST1387(CC1)
LTS018_Ia_ST1
SS1
2092071 bp
200 kbp
400 kbp
600 kbp
800 kbp
1000 kbp
1200 kbp
1400 kbp
1600 kbp
1800 kbp
2000 kbp
sdhA
adhP
tkt
alp3
glck
atr
MGE
PI-1
PheS
CPS
PI-2a
glnA

Figure S2. Comparison of representative Tn*916* and Tn*3872* sequences identified in our dataset and reference Tn*916* and Tn*917* from *Enterococcus faecalis*. Structure of LTS_Tn*916* and LTS_Tn*3872* aligned to Tn*916* and Tn*917* reference genomes from *E. faecalis* (GenBank: U09422.1, M11180.2). Tn*3872* is a Tn*916*-like MGE comprising Tn*917* flanked by left and right Tn*916* fragments. Names of most relevant genes involved in conjugation (orange), acquired AMR (blue), and recombination (red), can be found above and below alignments. Sizes of fragments denoted on the right size of each alignment. Sequence identity shown in grey ranging between 89-100%.

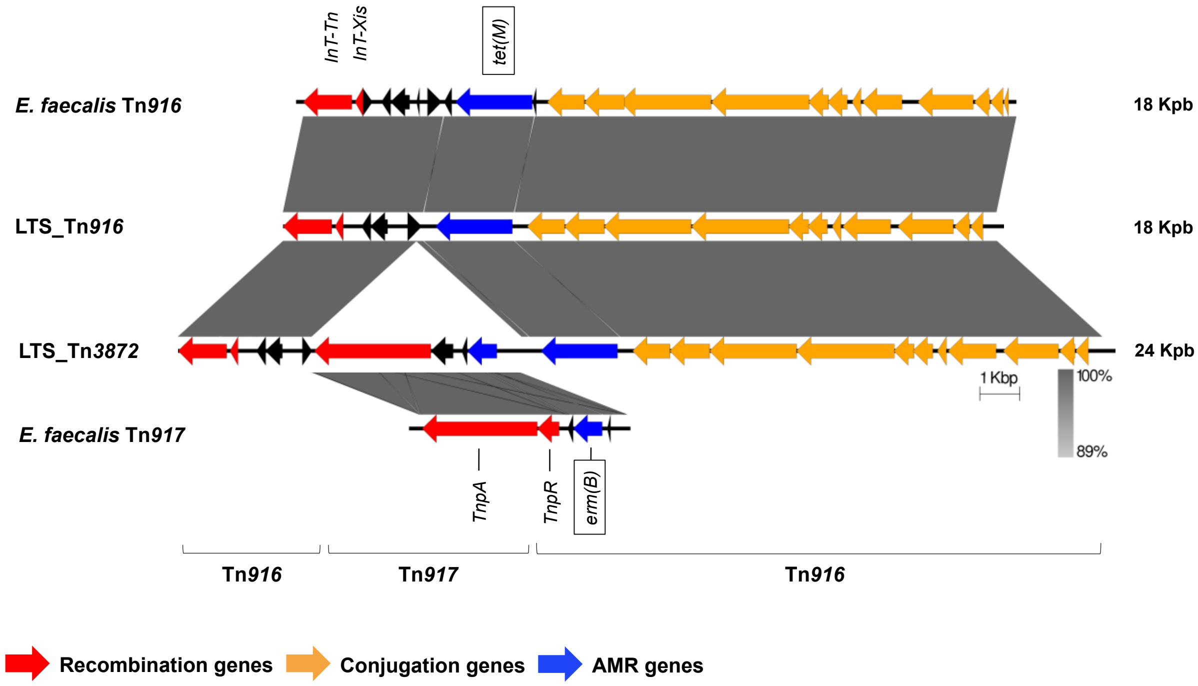

Supplement: Supplementary file 1 [file pathogens-11-01060-s001.zip › Supp_materials_final.pdf]
